# Sex differences, gonadal hormones and the fear extinction network: implications for anxiety disorders

**DOI:** 10.1186/2045-5380-2-3

**Published:** 2012-02-07

**Authors:** Kelimer Lebron-Milad, Mohammed R Milad

**Affiliations:** 1 Department of Psychiatry, Harvard Medical School & Massachusetts General Hospital, Boston, MA, USA

**Keywords:** menstrual cycle, sex hormones, estrogen, progesterone, estrus cycle, fear extinction

## Abstract

Convergent data from rodents and human studies have led to the development of models describing the neural mechanisms of fear extinction. Key components of the now well-characterized fear extinction network include the amygdala, hippocampus, and medial prefrontal cortical regions. These models are fueling novel hypotheses that are currently being tested with much refined experimental tools to examine the interactions within this network. Lagging far behind, however, is the examination of sex differences in this network and how sex hormones influence the functional activity and reactivity of these brain regions in the context of fear inhibition. Indeed, there is a large body of literature suggesting that sex hormones, such as estrogen, do modulate neural plasticity within the fear extinction network, especially in the hippocampus.

After a brief overview of the fear extinction network, we summarize what is currently known about sex differences in fear extinction and the influence of gonadal hormones on the fear extinction network. We then go on to propose possible mechanisms by which sex hormones, such as estrogen, may influence neural plasticity within the fear extinction network. We end with a discussion of how knowledge to be gained from developing this line of research may have significant ramifications towards the etiology, epidemiology and treatment of anxiety disorders.

## Background

There are now substantial data indicating that structural, cellular and molecular differences exist between the male and female brains in regions that are important for cognition, memory and affect, such as the hippocampus, amygdala and prefrontal cortex. Some of these differences may have clinical relevance, as marked disparities in disease incidence, manifestation, prognosis and treatment have been observed between the sexes. For example, men have a higher prevalence of conditions that emerge early in development, such as autism, attention deficit hyperactivity disorder and schizophrenia. Women, on the other hand, have a higher prevalence of disorders that emerge in adolescence or adulthood, such as major depression and anxiety disorders. Surprisingly, very little is known about the neural mechanisms that underlie the expression of sex differences in psychiatric disorders. A 2001 report by the Institute of Medicine highlighted the need to conduct scientific studies at the cellular, molecular and whole organism level that take into account sex as a variable to investigate the neural mechanisms that lead to epidemiological differences in psychiatric disorders.

**Figure 1:**
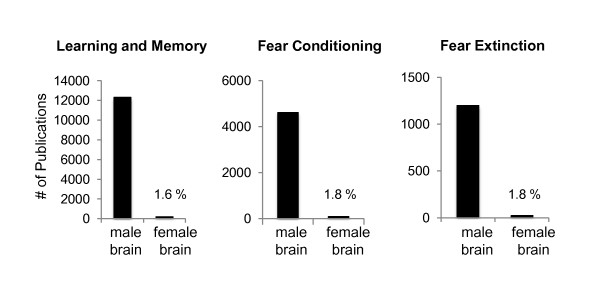
**Research studies using males vs females**.

The need to examine sex differences in the network mediating fear learning and its extinction can be surmised from two different perspectives. From a clinical perspective, we need to understand what contributes to the significant epidemiological differences in psychiatric disorders that are characterized by exaggerated fear and anxiety, such as post traumatic stress disorder. From a basic neuroscience perspective, it is essential that we understand how male and female brains differ in processing fundamental neurobiological phenomena such as emotional learning and memory. There is now a clear indication that failure in the function of brain regions that mediate fear learning and fear inhibition may be associated with the psychopathology of anxiety disorders [1-5]. Sexual dimorphism in the amygdala, hippocampus and medial prefrontal cortices is well documented [6,7]. These brain regions also contain elevated levels of estrogen receptors [8-10]. Thus, the structural and functional differences in these brain regions between the sexes may explain in part, or contribute to, some of the basic and clinical differences observed between men and women. In examining the literature pertinent to learning and memory, fear conditioning, and fear extinction, it is astonishing to note that of all that we have learned about these processes, less than 2% of this research has been focused on the female brain (Figure 1).

### The fear extinction network: a brief overview

Review of the fear extinction literature in general is beyond the scope of this article. We direct the reader to some recent reviews on fear extinction that focused on the translational aspects of this line of research [11], the general clinical relevance [12-16] and the molecular machinery of fear extinction [17]. Our review of fear extinction will be brief with the objective of setting the stage for the points we intend to make regarding sex differences in, and estrogen's influence on, the fear extinction network.

#### Components of the fear extinction network

Early studies of conditioned fear extinction showed that blockade of N-Methyl-D-aspartic acid (NMDA) receptors within the amygdala impair the extinction of fear potentiated startle [18]. A number of studies subsequently showed that lesion or pharmacological manipulations of the basolateral amygdala (BLA) and the intercalated GABAergic neurons within the amygdala interfere with fear extinction learning [19-21]. Electrophysiological data recorded from the BLA during fear extinction indicate the existence of two neural populations: one signals fear and the other signals fear inhibition or extinction [22]. Thus, in addition to its role in fear acquisition [23], the amygdala appears to also play a critical role in fear extinction [24,25].

In addition to the amygdala, the infralimbic (IL) region of the rat ventromedial prefrontal cortex (vmPFC) appears critical for the consolidation and retrieval of the extinction memory after a delay. Lesions [26-28], pharmacological manipulations [24,29,30] and electrophysiological recording [31] studies implicate the IL in extinction memory consolidation and expression. Moreover, electrical stimulation of IL simulates extinction memory [32,33]. Subsequent studies have further supported these findings using different experimental tools, including measuring intrinsic excitability of IL neurons [30,34,35], using metabolic mapping [36], potentiation of thalamic inputs to IL [37], and manipulations of hippocampal inputs to IL [38,39].

Another key structure that plays a role in fear extinction is the hippocampus. While its specific role in fear extinction is currently being investigated, it is likely that the hippocampus learns about the context in which fear learning took place and learns about the context in which extinction learning takes place [40,41]. During extinction recall, the hippocampus can, depending on the context, allow the expression of either the fear memory via activation of the amygdala fear neurons, or safety memory via activation of IL neurons [42]. A number of studies have now shown that the interaction between the hippocampus and IL during fear extinction is key to the success of extinction memory consolidation and expression [41,43-45].

Neuroimaging studies have translated these findings to the human brain using comparable fear conditioning paradigms, some of which use contextual manipulations of fear conditioning and extinction [46-49]. Imaging studies began by implicating the human amygdala in fear conditioning and also during extinction learning [46,50,51]. Recent functional magnetic resonance imaging studies (fMRI) designed specific paradigms to examine extinction recall and found that, like the rat IL, the human vmPFC increased activation to the extinguished cue during extinction recall; the level of activation positively correlates with the extinction recall magnitude [46,47,51]. Thickness of the vmPFC is also correlated with magnitude of extinction recall [52,53]. Like the amygdala and vmPFC, activation of the hippocampus during fear extinction is reported in a number of imaging studies during contextual conditioning [48] and extinction memory recall [47,51]. Deactivation of the hippocampus at the time of the delivery of the unconditioned stimulus (US) has also been reported in humans [54].

#### The molecular machinery mediating learning not to fear

There now exists a large database regarding the molecular machinery involved in fear extinction within the amygdala, hippocampus and IL in rodents. In BLA, interfering with mitogen activated protein kinase (MAPK), phosphoinositide 3-kinase (PI3-K), immediate early genes cfos and early growth response protein 1 (EGR-1) prevented consolidation of extinction [17,24,41]. Protein synthesis in BLA is also necessary for fear extinction [55]. Extinction training leads to structural changes in BLA. For example, mRNA for the brain-derived neurotrophic factor (BDNF) is up-regulated [56]. Furthermore, rats with lentiviral-induced reduction in BDNF receptors in the BLA can extinguish normally within a session, but were unable to recall extinction the following day, consistent with a role of BLA in consolidation of extinction [56]. In IL, extinction memory requires NMDA receptor activation [57,58], protein kinase A [17], MAPK [59], cannabinoid receptors [60], and protein synthesis [17,29]. In the hippocampus, extinction of context conditioning requires protein synthesis [61] cyclic adenosine monophosphate (c-AMP) [62], BDNF [43] and a number of protein kinases and their regulators [63].

### Sex differences during conditioning and its extinction

A number of studies have investigated differences between females and males in learning and memory using a number of behavioral tasks. For example, females acquire eye-blink conditioning at a faster rate relative to males [64]. In inhibitory avoidance tasks, females outperform males in escaping during a one-way avoidance task and a two-way avoidance task [65]. Studies conducted to examine sex differences in the acquisition of cued and contextual fear conditioning showed that male rodents exhibit increased contextual and cued fear conditioning relative to females [66-70], whereas other studies failed to show sex differences in these learning tasks [71].

Relative to fear acquisition, few studies have investigated potential sex differences during extinction learning and recall [72,73]. We showed that sex differences in fear extinction are influenced by the phase of the estrous cycle in female rats and the menstrual cycle in women [74-76]. When not taking cycle phase into consideration, differences in fear extinction recall were not noted in either female rats or in naturally cycling women. When females were divided into low and high endogenous estradiol groups, however, sex differences emerged. During extinction recall, male rats showed comparable levels of extinction retention to female rats with high estradiol; both were significantly higher than females with low estradiol [75]. The same pattern of results was observed in women. That is, men's extinction retention was comparable to that of women with high estradiol; and both groups showed significantly higher levels of extinction retention compared to women with low estradiol [76]. Thus, the lack of sex differences reported in previous studies or the discrepant results between studies may be the result of not taking into consideration the cycle phase of the animals being tested. These data also raise the following question: could sex hormones influence the learning, consolidation and plasticity typically associated with fear extinction in the female brain?

### Sex hormones influencing fear extinction in the female rat and in women

The data reviewed above indicate that sex hormones in rodents and humans may contribute to differences in fear learning and fear extinction. In support of this, estrogen treatment in ovariectomized female rats enhanced the acquisition of fear conditioning [70,77] and fear potentiated startle [78]; though elevated estrogen levels have been associated with reduced contextual conditioning in another study [67]. A few studies have shown that estrogen facilitated the extinction of passive avoidance [79] and conditioned taste aversion [80]. To date, there are few published studies that specifically examined the role of sex hormones on fear extinction learning and its subsequent recall. One exception is Chang *et al*., (2009), who have recently shown that infusion of estrogen into the hippocampus facilitates extinction of context conditioning and enhances hippocampal long term potentiation (LTP) [81].

**Figure 2:**
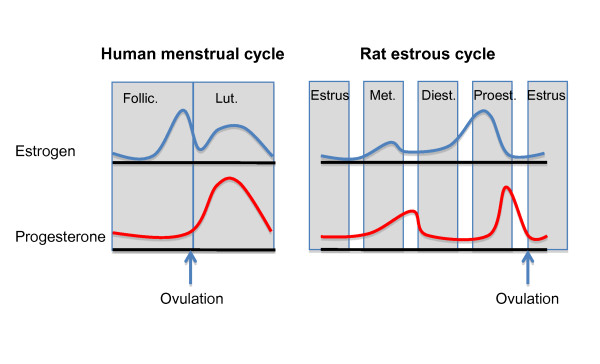
**The menstrual cycle and the estrous cycle **[164].

**Figure 3:**
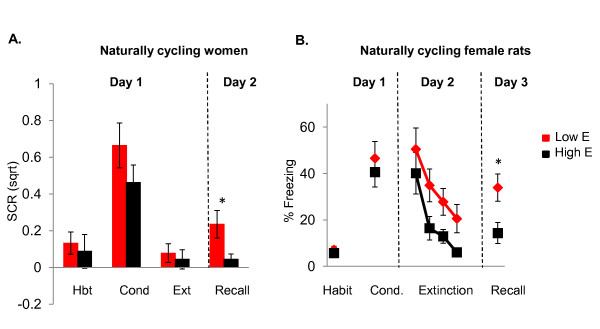
**Effects of endogenous estrogen on extinction recall**.

**Figure 4:**
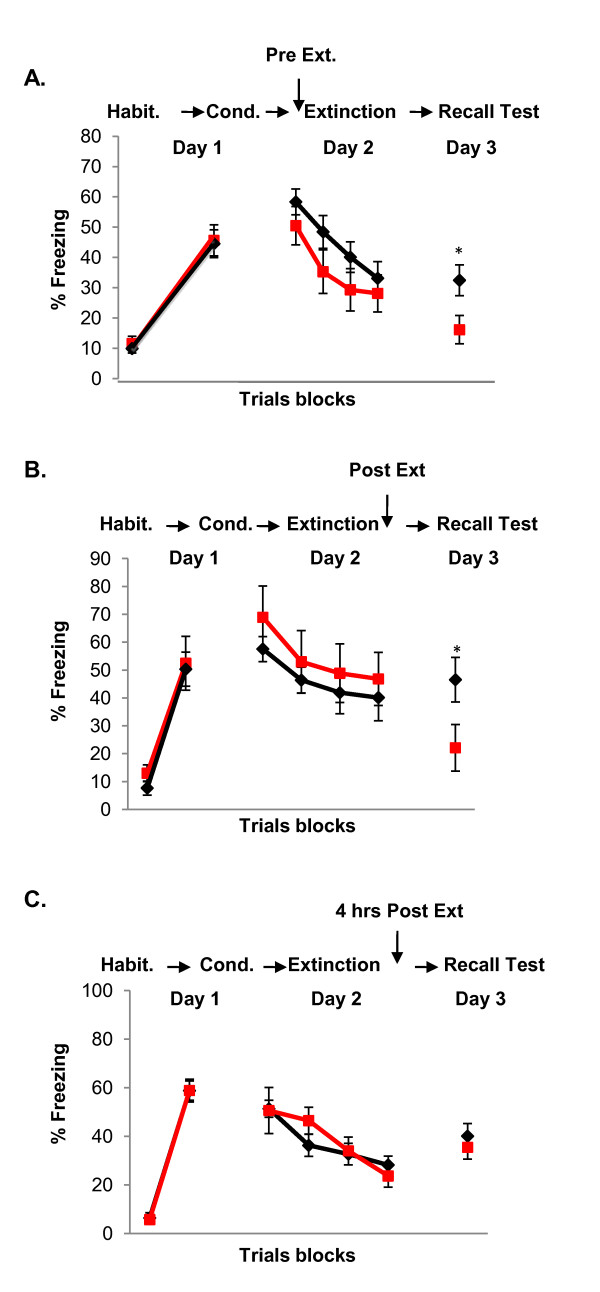
**Effects of exogenous estrogen on extinction recall**.

We have recently conducted a number of experiments examining how endogenous fluctuations and exogenous manipulations of sex hormones, particularly estrogen and progesterone, influence fear extinction in female rats. A diagram of the natural fluctuations of sex hormones during the estrous cycle in rats and the menstrual cycle in women is shown in Figure 2. We observed that naturally cycling females exhibited the least amount of freezing during extinction recall when they underwent extinction learning during the proestrus phase (high estrogen and progesterone) of the estrous cycle [75]. These findings suggest that estrogen and/or progesterone may facilitate the consolidation of extinction learning (Figure 3B). In support of this, systemic pre- or post-extinction administration of estradiol into female rats undergoing extinction learning in the metestrus phase (low estrogen and progesterone) significantly reduced freezing during recall (Figure 4a, b) [75]. Systemic blockade of estrogen receptors alpha (ERα) and estrogen receptor beta (ERβ) in female rats undergoing extinction in the proestrus phase significantly increased freezing [75]. In a later study, we observed that administration of ERβ but not ERα agonists were able to facilitate extinction recall in female rats undergoing extinction training in the metestrus phase of the cycle [74]. These data provide strong evidence that cycling sex hormones, estradiol in particular, in female rats do indeed influence extinction consolidation, possibly via the selective activation of the ERβ receptors.

**Figure 5:**
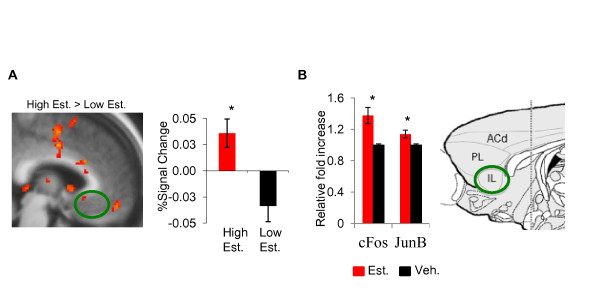
**Effects of estrogen in vmPFC activation and gene expression during extinction recall**.

In women, neuroimaging studies have shown that measures of fear and arousal are associated with changes in hormonal levels throughout the menstrual cycle [67,82,83]. Interestingly, increased vmPFC activation was observed in women in the luteal phase relative to those in the early follicular phase of the menstrual cycle while performing a Go-No-Go Task [84], suggesting that estrogen may facilitate the functional activation of the vmPFC. We have conducted an initial psychophysiological study to assess the influence of the menstrual cycle phase on recall of fear extinction in healthy women, and found that natural fluctuations of gonadal hormones do modulate extinction recall [85]. Women with high estrogen exhibited significantly enhanced extinction recall (that is, less fear) relative to women with low estrogen levels [76] (Figure 3A). In a more recent study, we replicated the effect of facilitated extinction recall in women with high estrogen and found that the increased estrogen levels in these women is associated with increased vmPFC, hippocampal and amygdala activation during extinction recall (Figure 5), further supporting the idea that estrogen may be playing a critical role in extinction memory consolidation [74].

### How might estrogen modulate extinction recall?

#### Estrogen and its receptors

In females, estrogen is primarily produced by the ovaries, while in males testosterone is produce by the testis and then aromatized into estrogen [86]. Estrogen can also be synthesized in brain regions, such as the hippocampus [87]. The most potent circulating estrogen is 17β-estradiol and the most characterized ERs are ERα and ERβ [88]. These receptors belong to the nuclear receptor superfamily and can be localized in the nucleus as well as in the cytoplasm of the cell [88]. ERα and ERβ are coded by different genes but share similar DNA-binding and ligand-binding domains. Estrogen binds to either ERα or ERβ through its estrogen response element DNA binding site. These result in receptor dimerization and subsequence gene transcription [89].

#### Localization of the estrogen receptors

Both receptors are located throughout the rostral-caudal extent of the brain and spinal cord, including regions of the fear circuitry [90,91]. Previous studies have shown that expression of ERα and ERβ overlap in many brain regions, such as the bed nucleus of the stria terminalis, medial and cortical amygdaloid nuclei, periaqueductal grey and locus coeruleus [91]. However, differences in expression have also been shown where one type of receptor is either expressed alone or at higher concentrations relative to the other. For example, ERα is the predominant receptor expressed in the ventromedial nucleus of the hypothalamus while ERβ is most prevalent in the hippocampus [92]. ERα and ERβ are expressed in the amygdala while ERβ is mostly expressed in PFC [93-95]. Activation of these two types of receptors leads to different behavioral consequences. Accumulating evidence now indicates that selective ERβ agonists typically exert potent anxiolytic activity when animals were tested in a number of behavioral paradigms [91,96,97]. In contrast, selective ERα agonists were found to be anxiogenic and correspondingly increased the hormonal stress response [91].

#### Sex differences in the estrogen receptors

ERα and ERβ have similar distributions in male and female brains, although subcellular distributions of ER to the nucleus, cytoplasm, dendrites and nerve terminals have been reported to be different in male and female human hypothalamus [98]. Although the functional consequences of these differences remain to be determined, this could indicate differential effects of estrogen in the male versus female brains regarding cellular processes, such as neurite extension, synaptic plasticity and mitochondrial energy regulation via mitochondrial ERs. There is also evidence for sex differences in intracellular signaling, expression of co-regulatory proteins, and in the response of the brain ER/aromatase system to injury [88].

#### Estrogen-induced molecular and cellular changes

The influence of estrogen on molecular and cellular changes in the brain has been examined mostly in the hippocampus [99,100] and in the hypothalamus [88]. Hippocampal estrogen enhances synaptogenesis and long-term potentiation (LTP) [101], increases the formation of dendritic spines [102,103], increases cell proliferation [104], and increases neural excitability [105,106]. Estrogen-induced LTP in the hippocampus is mediated via synaptic transmission of NMDA receptors [107], specifically through the increased expression of the NR2B subunit [108].

Increased estrogen is also associated with increased BDNF expression in the hippocampus [109,110]. It is possible that estrogen-enhanced extinction memory consolidation may be mediated via increasing BDNF expression in the hippocampus or the vmPFC. Indeed, BDNF expression is critical for successful fear extinction in both rodents and humans [111]. Peters *et al. *(2010) showed that hippocampal infusion of BDNF before extinction training enhanced extinction memory in an NMDAr dependent process, suggesting that hippocampal BDNF modulates IL activity during the consolidation of extinction memory [112]. Estrogen and BDNF are known to have similar mechanisms of actions, activate the same cascades, and have the same behavioral effects, especially within the hippocampus. For example, both estrogen and BDNF enhance hippocampal-dependent learning [109,110]. The direct interaction between estrogen and BDNF during fear extinction has not yet been investigated.

Few studies have examined the influence of estrogen on the function of the vmPFC in the rat. Estrogen has been shown to increase spine density in vmPFC [113], preserve the functional integrity of the IL when subjecting the rats to chronic stress [114], and to enhance working memory [115]. These data indicate that estrogen influences the function of the vmPFC in the rat brain. Markers of neural activity and synaptic plasticity, such as c-Fos, Jun-b and BDNF, have been shown to be modulated by estrogen variance [74,116-118]. Therefore, we propose that estrogen may modulate fear extinction learning and consolidation via its interaction with a number of molecular markers of plasticity, within the fear extinction network.

We recently examined the influence of estradiol administration on c-fos and Jun-b mRNA expression within some components of the fear extinction network. Female rats undergoing fear extinction during the metestrus phase of the cycle (low estrogen) received estradiol immediately post-extinction training and were sacrificed immediately after a brief extinction recall test. The results of this study showed that administration of estradiol after extinction training enhanced c-fos and June-b mRNA expression in IL (Figure 5b) and significantly reduced the expression of both in the amygdala during recall [74]. These data further support the idea that estrogen fluctuations may be a critical modulator of extinction memory consolidation in females. In order to fully understand the role of estrogen in fear extinction, it is necessary to identify the molecular cascades by which estrogen may enhance extinction memory and investigate sex differences in estrogen action within the extinction network.

### What about progesterone and testosterone?

#### Progesterone

Several studies have shown that progesterone administration to ovariectomized female rats facilitates contextual and cued fear conditioning, and enhances cognitive performance in a variety of other behavioral tasks in mice [119]. In healthy young women, administration of progesterone during the early follicular phase (when progesterone and estrogen are at their lowest level) led to increased reactivity in the amygdala while looking at threatening faces [120]. Also, progesterone increased the functional coupling of the amygdala with the mPFC [121], indicating that progesterone influences interactions between the amygdala-PFC circuits. Moreover, progesterone is metabolized into allopregnanolone [122], which acts via GABA_A _receptors and appears to have anxiolytic effects when infused into the amygdala or vmPFC before an elevated plus-maze test and shock-probe burying test [122,123]. In other tasks, progesterone facilitates extinction of cocaine self-administration [124]. We have shown that systemic administration of progesterone into female rats (either alone or in conjunction with estrogen) facilitates extinction consolidation [75], suggesting that progesterone appears to also influence the function of brain regions involved in extinction consolidation. Our data gathered in women, however, showed that variance in progesterone levels in two separate cohorts of women did not correlate with extinction recall [74,76]. While progesterone's influence on fear extinction may differ across species, it is important to note that we were not able to fully examine the effects of progesterone independent of estrogen. Thus, it remains possible that progesterone may have an effect on fear extinction consolidation in women directly or perhaps by interacting with estrogen. Additional studies are needed to further examine the role of progesterone in fear extinction in women.

#### Testosterone

It is established that besides the masculinization/defeminizing role of testosterone during sexual differentiation, this hormone is also critical for the modulation of behavioral and physiological responses to anger [125,126]. Men demonstrating higher dominance express high levels of testosterone. In male primates, dominance is associated with higher testosterone levels and a decrease in stress response, indicating that dominant males find dominance signals less stressful and are more primed to engage in a dominance challenge [127,128]. More interestingly, previous studies have shown that testosterone reduces cortisol response and stress axis reactivity [129,130]. Other studies demonstrated that endogenous as well as exogenous testosterone influence neural reactivity to threatening faces in the amygdala and orbitofrontal cortex (OFC) in males, and in the amygdala in women [128,131]. Exogenous testosterone also increases amygdala reactivity to threatening faces, but reduces functional coupling between the amygdala and OFC in middle-aged women, suggesting that the testosterone may regulate interactions between amygdala and OFC [128]. It is important to note that effects of testosterone may be mediated via direct interactions with androgen receptors or via conversion to other steroids. Testosterone is metabolized to dihydrotestosterone (DHT), which also acts via androgen receptors, and to androstanediol that modulates GABA_A _receptor similar to allopregnanolone [132]. Lastly, testosterone is also aromatized into 17b-estradiol, the most potent type of estradiol, and it has been suggested that most of the effects of testosterone are mediated by estrogen [133,134]. Clinical and basic studies are needed to assess the role of testosterone during fear extinction.

### Clinical relevance

Increased expression of inappropriate fear is the hallmark of anxiety disorders [135-138]. A large body of evidence from neuroimaging studies indicates that the neural circuits subserving fear conditioning and extinction are impaired across the different anxiety disorders [12]. For example, post-traumatic stress disorder (PTSD) patients exhibit decreased vmPFC and hippocampal activation along with exaggerated amygdala activation during the processing of emotional stimuli in a wide array of paradigms [139-142]. Experimental extinction is also deficient in PTSD patients [10,143-146]. We have recently shown that fear extinction is deficient in PTSD patients and that such deficiency is associated with aberrant activation of the vmPFC, hippocampus and amygdala (in addition to insula and striatal regions) during fear acquisition, extinction learning and extinction recall [1,5,54,74].

Epidemiological data suggest that the prevalence of anxiety disorders is higher in women relative to men. Women are more likely to develop panic disorder (8% vs. 3%), PTSD (12.5% vs. 6%) and generalized anxiety disorder (GAD) (7% vs. 4%) (Pigott, 2003; Breslau *et al*., 1998;Kinrys and Wygant, 2005). Aside from prevalence, women diagnosed with PTSD have longer symptom duration (48 vs. 12 months) [147], have higher symptom severity and functional impairment [148], and have worse quality of life [149]. Women diagnosed with GAD are more likely to develop comorbid psychiatric disorders and have worse prognoses and impairments [150]. In addition to increased prevalence of panic disorder in women, studies also suggest that panic attacks occur more frequently in women relative to men [151]. These findings point to brain-based differences in the processing of emotional stimuli in women compared to men, and suggest that sex hormones, such as estrogen, play a key role in mediating these differences. Indeed, there are some clinical data suggesting that estrogen therapy improves anxiety symptoms in postnatal depression [152], in recurrent postpartum affective disorder [153], and in menopause [154,155]. Despite these glaring differences, it is puzzling that so few studies have considered sex differences as a critical variable of interest.

## Conclusions

The data reviewed herein point to clear sex differences in, and clear influence of, sex hormones, especially estrogen, on fear extinction. The data discussed show that sex hormones may have direct effects on the molecular machinery mediating synaptic plasticity in the hippocampus, and the vmPFC during fear extinction. These data also point to significant clinical implications. Future studies need to develop paradigms to test specific hypotheses based on what we know thus far. For example, does estrogen serve a protective function against elevated fear and anxiety? Could transient periods of low estradiol levels be associated with impaired retention of safety memory? In addition, fear extinction in women using oral contraceptives and in menopausal women with and without hormone replacement therapy should be examined. This is especially important given that the use of oral and intrauterine contraceptives is known to reduce endogenous cycling estradiol levels [156]. Moreover, women appear to be vulnerable to developing mood and anxiety disorders during postpartum [157,158] and menopausal periods [159-163] when endogenous estradiol levels are low. Additional studies investigating the effects of cycling sex hormones and exogenous manipulations of these hormones in animal models of fear inhibition could potentially introduce ways to adapt, improve or produce therapies specifically tailored to women.

## Abbreviations

BDNF: brain-derived neurotrophic factor; BLA: basolateral amygdala; cAMP: cyclic adenosine monophosphate; DHT: dihydrotestoterone; EGR-1: early growth response protein 1; ERα: estrogen receptors alpha; ERβ: estrogen receptor beta; fMRI: functional magnetic resonance neuroimaging; GAD: generalize anxiety disorders; IL: infralimbic; ITC: intercalated GABAergic neurons; LTP: long-term potentiation; MAP kinase: mitogen activated protein; NMDAr: N-Methyl-D-aspartic acid receptor; OFC: orbitofrontal cortex; PI 3-kinase: phosphoinositide 3-kinase; PTSD: posttraumatic stress disorders; US: unconditioned stimulus; vmPFC: ventromedial prefrontal cortex

## Competing interests

The authors declare that they have no competing interests.

## Authors' contributions

KLM and MRM contributed equally to this manuscript. Both authors read and approved the final manuscript.

## Authors' information

N/A

## Endnotes

There are no endnotes for this manuscript.
